# Rethinking Medical Spanish Competence: Language Proficiency, Clinical Communication, and Inclusion

**DOI:** 10.2196/110302

**Published:** 2026-09-24

**Authors:** Juli McCarroll

**Affiliations:** 1 Department of Medical Library, Western Michigan University Homer Stryker M.D. School of Medicine, 1000 Oakland Drive, Kalamazoo, MI, 49008, United States, 1 269-337-6116

**Keywords:** medical Spanish, inclusive communication, LGBTQ+ health, bias, clinical communication, language concordance, standardized patients, simulation training, digital education, curriculum development

I commend Lopez Vera [1] for her patient-informed framework integrating inclusive, bias-aware communication with Spanish-speaking lesbian, gay, bisexual, transgender, queer, plus (LGBTQ+) patients as a clinical skill in medical Spanish education. The tutorial appropriately moves beyond vocabulary acquisition by incorporating patient-informed communication priorities, curricular design principles, and integrated learning objectives. This framework also creates an opportunity to address a broader question: what does it mean for a learner to be competent to provide clinical care in Spanish?

As an LGBTQ+ community member, I view competence more broadly. A clinician may possess substantial Spanish proficiency but communicate in ways that make an LGBTQ+ patient feel unseen or unsafe. Conversely, a learner may understand inclusive terminology but lack the Spanish proficiency necessary for complex clinical encounters. These are different competencies, and patients experience the consequences of both.

The emerging qualified multilingual assessment (QMA) framework described by Molina et al [2] and Biswas et al [3] offers an important complement to this discussion. The QMA was developed to assess oral language proficiency for direct clinical care rather than for qualification as a medical interpreter. Importantly, demonstrating proficiency does not eliminate the need for interpreter services when a clinical situation exceeds a clinician’s demonstrated language abilities. This distinction is particularly relevant for inclusive medical Spanish education.

A learner may demonstrate inclusive communication behaviors, such as asking a patient for their preferred form of address, avoiding heteronormative assumptions, and using open-ended relationship language, without possessing sufficient Spanish proficiency for independently conducting a complex clinical encounter. Conversely, a learner may demonstrate substantial Spanish proficiency while communicating in ways that are linguistically accurate but clinically exclusionary. Lopez Vera’s [1] framework recognizes this latter possibility, emphasizing that inclusive communication requires more than substituting terminology and involves adapting language to patients’ preferences.

These observations suggest that language proficiency and inclusive clinical communication should be assessed as complementary, rather than interchangeable, competencies. This raises several questions for medical educators. Should readiness for language-concordant care require a defined level of clinical language proficiency and demonstrated competence in patient-centered, bias-aware communication? Should assessments distinguish between knowing inclusive terminology and sustaining inclusive communication while obtaining a history, explaining a diagnosis, counseling a patient, and responding to unexpected clinical information? Most importantly, should curricula explicitly teach learners to recognize when their linguistic limitations warrant professional interpretation support?

I propose a three-dimensional model of medical Spanish competence that encompasses clinical language proficiency, clinical communication competence, and inclusive communication competence. This model could help educators determine not only whether a learner speaks Spanish but also what the learner can safely do in Spanish, what additional development is needed, and when interpreter support remains appropriate. This distinction is particularly important because speakers of languages other than English may bear additional responsibility for determining whether their language skills are sufficient for a particular clinical task.

Lopez Vera’s [1] tutorial provides an important foundation for patient-informed, inclusive medical Spanish education. Integrating this framework with emerging approaches to multilingual proficiency assessment could move the field toward a more precise and clinically meaningful definition of language-concordant competence.

## Abbreviations

LGBTQ+: lesbian, gay, bisexual, transgender, queer, plus
QMA: qualified multilingual assessment
